# Health behaviour in Germany – ongoing cause for concern!

**DOI:** 10.25646/10289

**Published:** 2022-09-14

**Authors:** Julika Loss

**Affiliations:** Robert Koch Institute, Berlin Department of Epidemiology and Health Monitoring

The health risks associated with overweight and obesity as well as their worldwide increasing prevalence have been known for decades. Before the term ‘pandemic’ became synonymous with the spread of COVID-19 to the public, the terms ‘obesity pandemic’ or ‘adiposity epidemic’ were widely discussed in the media. Did the high awareness for the topic of overweight also lead to a turnaround in the pervasiveness of overweight and adiposity? Schienkiewitz et al. analyse data from the nationwide survey GEDA (German Health Update) from 2019 and 2020, and the results are sobering: Compared to earlier surveys from 2012, they show a consistently high prevalence of overweight (approximately 47% of all women, 61% of all men), a slight increase to 19% for both sexes has even been reported in the case of adiposity. The news is thus still worrying. This is all the more alarming as the COVID-19 pandemic has tragically highlighted the particular vulnerability of people affected with overweight and adiposity – an excessive BMI represents a risk factor for severe illnesses among people who got infected with SARS-CoV-2. ‘Heavy Times’ is used as the title of an article in the magazine ‘Die ZEIT’ from July of 2021, and further: ‘Where overweight and obesity are widespread, particularly large numbers of people die during the Corona crisis.’ In addition, there are observations that the mitigation measures implemented in the pandemic have promoted the increase of overweight in large sections of the population. The COVID-19 pandemic thus magnifies that not only a continuous nationwide monitoring of the BMI is necessary, but also the prevention and treatment of overweight and obesity.

Lifestyle plays an important role in the development of overweight, with a poor balance between energy intake and expenditure being critical for weight gain. In the life of many people, intensive sports regimen come short, but also low-impact physical activity is missing from daily routines, such as riding a bike, walking, or climbing stairs. At work, during leisure time, and even when covering distances, people mostly sit – at the table, on the couch, or in the car. Our society is characterised by a sedentary lifestyle. In recent years, prolonged periods of sitting have been described as independent risk factor for the development of various chronic diseases. The negative results of sitting can most likely only be compensated by extremely long periods of physical activity during the day. This is reason enough to add the question about time spent sitting in the GEDA 2019/2020-EHIS survey for the first time. Manz et al. show that every day, people in Germany cumulate a significant amount of time that is spent in the sitting or lying position – in addition to sleeping. 17% of women and 22% of men sit or lie more than eight hours a day. What is unusual about this: In contrast to most of the other health-related risk factors, such as tobacco smoking or poor diet, this particularly affects the higher education groups. This may be due to the fact that certain professions or clerical work are represented more frequently in higher education groups. There are no suggestions yet with regard to an upper limit or a maximum ‘dose’ for periods of sitting. It can nonetheless be assumed that a significant proportion of the adult population in Germany endangers its own health because of long periods of sitting, combined with little movement. This is why the awareness that sitting is a health-related risk factor must be strengthened – among the general population as well as among those professionals, who are responsible for designing learning and working environments. When spending time at the office, at university or at school, people have to be given the opportunity to stand (up), to interrupt periods of sitting, to work while standing, as well as to design their breaks in an active and mobile manner.

Contrary to the comparatively ‘new’ risk factor of sitting, the substantial health risks of tobacco smoking are well-known to almost all people, and have been for many years. However, the smoking rate in Germany is still high, as reported by Starker et al. in their Focus article in this issue: Approximately a third of all men and a quarter of all women smoke at least occasionally. Especially in the low education group, the percentage of smokers is higher. Add to this 8% of the non-smoking population affected by passive smoking. The goal that less than five percent of adults consume tobacco products by the year 2040 is still far away. The ‘Strategy for a tobacco-free Germany 2040’ published last year set this ambitious target. It also names ten concrete political measures, including partial steps for their implementation, to make cigarette smoking more difficult and to promote quitting smoking. There is in fact still significant leeway in the implementation of measures. Even though the inception of the Non-Smoker’s Protection Act 2007 was an important milestone in the German tobacco control policy, Germany nonetheless lags behind other European countries in the implementation of regulatory measures. The Corona pandemic also shows an effect on the tabaco consumption: On the one hand, there are smokers who reported having used the pandemic to quit smoking. On the other hand, there is a proportion of smokers who report they consume even more tobacco than before – possibly promoted by pandemic-related mental stress and worries or enhanced by working from home. This is one more reason not to neglect smoking as the most important behavioural risk factor to health.

The data in this issue show an unabatedly urgent need for action for prevention in order to mitigate the important risk factors for malignant and cardiovascular diseases. Regardless of whether dealing with maintaining normal weight, avoiding prolonged sitting, or not smoking: What is crucial is to design the environments and settings in Germany in such a way that protecting his or her health is made easy for everyone.

